# Comparison of GTR, T-PRF and open-flap debridement in the treatment of intrabony defects with endo-perio lesions: a randomized controlled trial

**DOI:** 10.4317/medoral.23231

**Published:** 2019-12-24

**Authors:** Gülbahar Ustaoğlu, Zeliha Uğur Aydin, Ferhat Özelçi

**Affiliations:** 1 Assistant Professor, Department of Periodontology, Faculty of Dentistry, Bolu Abant İzzet Baysal University, Bolu, Turkey; 2 Assistant Professor, Department of Endodontics, Faculty of Dentistry, Bolu Abant İzzet Baysal University, Bolu, Turkey; 3 Research Assistant, Department of Periodontology, Faculty of Dentistry, Bolu Abant İzzet Baysal University, Bolu, Turkey

## Abstract

**Background:**

Titanium- prepared platelet rich fibrin (T-PRF) is an autologous hemo-component with a high concentration of platelets that also incorporates leukocytes, and growth factors into the dense fibrin matrix and can be used as a healing biomaterial. This study assesses the adjunctive use of T-PRF in intrabony defects (IBDs) with open flap debridement (OFD) in comparison with guided tissue regeneration (GTR) as a gold standard and OFD alone as a control.

**Material and Methods:**

A total of 45 patients (15 per group) were randomized as either T-PRF (test group), GTR (test group), or OFD alone (control group) sites. Probing depth (PD), clinical attachment level (CAL), and IBD were recorded. The radiographic depth of IBD was also measured. Primary outcomes assessed were changes in PD, CAL, and radiographic IBD that were assessed at the beginning and nine months later.

**Results:**

The PRF and GTR group showed significant improvement in clinical parameters compared with the OFD alone (control group) at nine months. While there were no significant differences in PD and CAL between test groups (T-PRF and GTR groups), the significant difference was found in radiographic IBD depth.

**Conclusions:**

T-PRF may give similar successful results as GTR in the treatment of IBDs with endo-perio lesions.

** Key words:**Flap surgery, guided tissue regeneration, intrabony defects, periodontitis, root canal therapy.

## Introduction

Pulp tissue may be affected by several causes, including trauma, periodontal disease, caries, restorative procedures, restorative materials, and chemical or thermal damage. The periodontium and pulp have a relationship at embryonic, anatomic, and functional levels. For this reason, through several pathways, horizontal and/or vertical cross-infection may develop between the root canal and the periodontal tissue, including apical foramen, lateral and accessory canals, dentinal tubules, palatal grooves, iatrogenic root canal perforation and root fracture (1). The vitality of the pulp can be affected by deep periodontal pockets extending to the apex of the tooth, leading to damage that may range from hyperemia to necrosis. This damage may result in an endo-perio lesion (2).

Endo-perio lesions may raise difficulties in terms of correct diagnosis and prognosis. Simon *et al*. (3) classified endo-perio lesions into five subgroups as follows: primary endodontic diseases, primary periodontal diseases, and combined disease including primary endodontic disease with secondary periodontal involvement, a primary periodontal disease with secondary endodontic involvement and true combined disease. This classification provides a valuable guide for clinicians to achieve the correct diagnosis and clinical approach. Finding out the etiology of the lesions is essential in determining the right treatment.

Primary periodontal disease with secondary endodontic involvement and true combined endodontic-periodontal diseases need both endodontic and periodontal treatments (4). The severity of periodontal disease and the response to periodontal treatment play an important role in the prognosis of these cases. Conventional endodontic and periodontal treatments may be insufficient to stabilize the tooth in these cases when bone loss is excessive. Therefore, resective and regenerative treatment options are to be considered (5). Today, intrabony defects (IBDs) can be treated through various treatment approaches, including guided tissue regeneration (GTR) using barrier membranes, various types or a combination of grafting materials, enamel matrix proteins, and autologous platelet concentrates (6,7).

GTR yields successful results in that the epithelial cell migration to the defect area is prevented with various membranes with or without bone graft materials. The bone graft materials then form a scaffold for the resident cells of the host, promoting either osteoinductive or osteoconductive pathways (8). Another technique found to be successful in the management of IBDs is use of autologous platelet concentrate to obtain healing biomaterials. Platelet-rich fibrin (PRF) is a bioactive scaffold used to achieve the regeneration of periodontal soft and hard tissue. Wound healing begins with the formation of a fibrin clot, platelet adhesion, and aggregation, followed by the release of many growth factors by the alpha granules of platelets, including platelet-derived growth factor (PDGF), vascular endothelial growth factor (VEGF) and transforming growth factor (TGF)-α and -β. Hence, these growth factors promote fibroblast chemotaxis, proliferation, contraction, extracellular matrix deposition, and re-epithelialization in wound healing (9). Titanium-prepared platelet-rich fibrin (T-PRF) is a new platelet concentrate found to be more effective than glass tubes in activating platelets thanks to its thicker fibrin texture and longer resorption time in the tissues (10). Various treatment options including gingival recessions, IBDs, sinus lifting procedure, and periodontal healing have made use of T-PRF (11,12).

In the literature, no studies comparing the efficacy of GTR and T-PRF in the treatment of endo-perio lesions have been found. Thus, this study aimed to examine the clinical and radiographic effectiveness of the GTR and T-PRF and open flap debridement (OFD) in the treatment of IBDs with endo-perio lesions.

Materials and Methods

This study included 45 patients (systemically healthy, non-smokers; 22 females and 23 males; age range: 26–59 years, mean ± SD: 40 ± 8.37 years) with IBDs associated with primary periodontal lesion with secondary endodontic involvement or true combined endodontic-periodontal lesions in single-rooted teeth. This 9-month study was designed as a double-blinded, single centered, prospective randomized controlled clinical trial. Approval was obtained from the clinical research ethics committee of Bolu Abant İzzet Baysal University (no: 2018/81), and it was conducted in compliance with the Declaration of Helsinki (1975, revised in 2000). Approved informed consent forms were provided by all patients before their participation in the study. 

The inclusion criteria were as follows:

1. Presence of interproximal probing pocket depth ≥ 5 mm with two- or three-wall IBDs ≥ 3 mm deep after root canal therapy and non-surgical periodontal treatment

The exclusion criteria were as follows:

1. Smoking

2. Pregnancy or lactation

3. Being immunocompromised

4. Poor oral hygiene

5. Systemic problems affecting periodontal tissues and blood coagulation

6. One-walled defect sites after flap reflection

- Pre-surgical Treatment

Root canal treatment and initial periodontal therapy were performed simultaneously. After local anesthesia was administered (Ultracaine DS Forte, Aventis Pharma, Istanbul, Turkey), rubber-dam was used to isolate the teeth. After preparation for the traditional endodontic access cavity, the apical patency was checked by using the 10 K type file (Dentsply Maillefer, Ballaigues, Switzerland). Using an apex locator [The Gold Reciproc Motor (GRM), VDW GmbH, Munich, Germany], root canal length was determined to be 1 mm shorter than apical foramen. All root canals were prepared by using 15 and 20 K type files (Dentsply Sirona). For preparations of root canals, we used the Protaper Universal F1, F2, F3, or F4 (Dentsply, Ballaigues, Switzerland) files. During preparation, 2 ml of 5.25% NaOCl (Wizard, Rehber Chemistry, Istanbul, Turkey) was used after each filing of each canal. Two milliliters of 17% EDTA (Wizard, Rehber Chemistry) was used for the final irrigation in order to remove the inorganic components of debris and open the dentinal tubules. The paper point was used to dry the root canals. The root canals were obturated by using a sealer (Dentsply, DeTrey, Konstanz, Germany) and ProTaper Universal F 4 gutta-percha (Dentsply Maillefer) according to the single cone technique. At last, the teeth were restored with the use of composite resin.

Simultaneous with root canal treatment, all patients had undergone a full-mouth supragingival and subgingival scaling and root planning (SRP) procedures and were given careful instructions regarding proper oral hygiene. Six weeks after the phase I therapy, a periodontal reassessment was performed. The defect sites were randomly divided into three groups with the aid of digital software (random.org.) (in each group, 15 defect sites were evaluated), OFD alone group, T-PRF group, and GTR group.

The allocation of the groups was concealed from each participant throughout the study to avoid bias. All periodontal therapies and surgical procedures were performed by the same operator (G.U.). Clinical parameters were carried out by another operator (F.Ö.) blinded to the groups.

- Clinical parameters

1. Probing depth (PD): Distance from the gingival margin to the base of the pocket

2. Clinical attachment level (CAL): Distance from the cementoenamel junction to the base of the pocket 

3. Site-specific plaque index (SPI) (13) 

4. Modified sulcus bleeding index (mSBI) (13).

**Figure 1:**
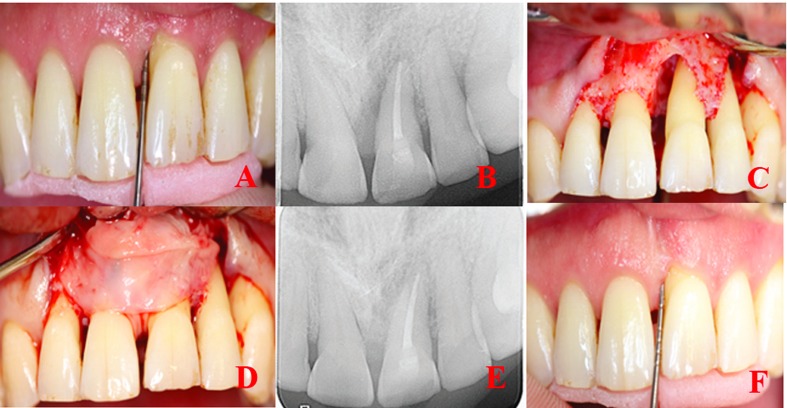
T-PRF procedure in the treatment of an intrabony defect with endo-perio lesion. A) Preoperative clinical view, B) Baseline radiograph, C) Intraoperative clinical image of typical intrabony defect, D) The defect is filled with the T-PRF membrane, E) Nine months postoperative radiograph, F) Nine months post-operative clinical view.

PD and CAL measurements were recorded before periodontal surgery and at six aspects per tooth by using customized acrylic stents with grooves providing reproducible placement of Williams periodontal probe (HuFriedy, Chicago, IL, USA) (Fig. 1). All clinical parameters were recorded before periodontal surgery and after nine months.

- Radiographic parameters

All periapical radiographs were obtained by using the same X-ray device (Carestream CS 2100) and photostimulable phosphor (PSP) plates with a holder. The long-cone parallel technique was used. The same scanner (Dürr Vista Scan Mini View) was used to scan all images. The previously described computer-aided program (Image J, Maryland, USA) was used for the measurement of the radiographic IBD depth (vertical distance from the crest of the alveolar bone to the base of the defect)(14). Radiographic images were taken at the baseline and the 9th postoperative month.

- Preparation of T-PRF

Blood samples were collected from the antecubital vein of the right or left arm of the patient at the first attempt and drawn into grade IV sterile titanium tubes immediately. The tubes were then centrifuged for 12 minutes at 2800 rpm and room temperature. Following centrifugation, sterile tweezers were used to remove the T-PRF clots from the tubes. Then T-PRF clots were separated from the red blood cell base and left on sterile woven gauze.

- Surgical treatment

**Figure 2:**
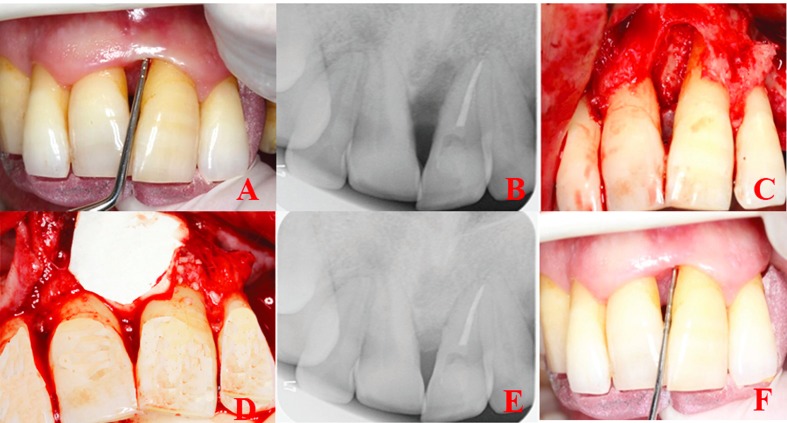
GTR procedure in the treatment of an intrabony defect with endo-perio lesion. A) Preoperative clinical view, B) Baseline radiograph, C) Intraoperative clinical image of typical intrabony defect, D) The defect is filled with allograft and covered with a resorbable membrane, E) Nine months postoperative radiograph, F) Nine months of post-operative clinical view.

Patients with a full mouth dental plaque score below 1 and a test site plaque score of 0 underwent a surgical procedure (15). Pre-surgical extra-oral and intra-oral antisepsis were provided with the use of iodine and 0.12% chlorhexidine solutions. Double flap approach was chosen in all cases (16). Sulcular incisions were performed both buccally and lingually following topical and local anesthesia. Flaps were extended horizontally (mesially and distally) to obtain complete access to the IBD. An incision is made at the buccal aspect of the interdental papilla overlying the intraosseous defect. A microsurgical periosteal elevator was used to raise a flap on both buccal and oral sides. The defects were cleaned from granulation tissues before root planning with area-specific curettes (Gracey curettes, Hu-Friedy). Then, saline was used to rinse the exposed root surface. Any material was not applied to the defects of the control group. Group sites were treated with OFD by using T-PRF only in the first test. Compressed T- PRF membranes were adapted over the defects (Fig. 1), and they were treated with OFD by using allograft (Maxxeus Dental, Kettering, OH, USA) + collagen membrane (Collagene AT, Padova, Italy) in the second test. The hydrated graft was placed tightly into the defects at the level of the surrounding bony walls. Collagen membranes with proper size were positioned to cover the grafted area and the adjacent 2–3 mm of bone tissue (Fig. 2). The flaps were then repositioned with 4-0 non-absorbable, monofilament, and polypropylene suture (Propylene, Doğsan, Trabzon, Turkey).

- Postoperative care

All patients were prescribed the same antibiotics during the postoperative period (1000 mg amoxicillin + potassium clavulanic acid after checking medical history for allergies, twice a day for seven days), anti-inflammatory analgesics (25 mg dexketoprofen, 3 times a day) and chlorhexidine digluconate rinses (0.12%) twice a day for two weeks. Sutures were removed on the 10th postoperative day (12). Patients were advised to use a soft toothbrush to brush the operative site. All patients were reinstructed in terms of proper oral hygiene measures in the 6th postoperative week. They were followed once a week for the first postoperative month and again at the third month for information on operation site and professional plaque removal if needed.

- Statistical Analysis

A power analysis was performed based on a previous study (17), and a sample size of 15 defects per-protocol group was sufficient to detect a clinically significant mean difference in percent decrease from initial to final measurement of the CAL level ( alpha level = 0.05, 80% power, and effect size = 1.0) (G*Power 3.1 software, Heinrich Heine University, Dusseldorf, Germany). Statistical analyses were performed using the SPSS software (SPSS Inc., Chicago, IL). Results were considered statistically signiﬁcant if below 0.05. The results were averaged (mean ± standard deviation) for the clinical and radiographic parameters. The Wilcoxon matched-pairs signed-rank test was used to analyze the changes between the baseline and the 9th-month values for all groups. For comparisons between groups, the Mann-Whitney U-test was used to compare clinical and radiographic outcomes between the baseline and the 9th-month values. The x2 test was used to compare mSBI and PI scores.

## Results

Intra-examiner calibration was performed by examining the 25 sites twice with 24 hours before the study. Measurements were accepted to be reproduced within a 1.0 mm difference and at 90% level at the baseline and 24 hours later. The correlation coefficient for the assessment of intra- examiner reliability was 0.91.

The study was completed with 45 patients without dropout. Table 1 shows the changes between the groups in terms of clinical and radiographic measurements at the baseline and the 9th postoperative month. A statistically significant decrease was found in PD, CAL and IBD depth in all groups (*p* < 0.05). No statistically significant difference was found between the T-PRF and GTR groups in terms of the baseline and the 9th-month measurements of PD and CAL, which were found to be significantly higher than the control group (*p* < 0.001). The decrease in IBD depth was obtained in the T-PRF (2.97 ± 0.77), GTR (3.85 ± 1.16) and the control groups (0.9 ± 0.80), and the differences between the groups were statistically significant (Table 1). A statistically significant decrease was found between the baseline and the 9th-month measurements of all groups in terms of plaque and sulcus bleeding indices (*p* < 0.001) (Table 2).

**Table 1:**
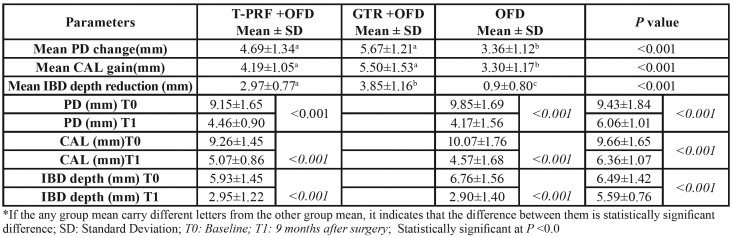
Changes (mean ± SD) in clinical and radiographic parameters between groups over a 9-month period, and clinical and radiographic parameters in groups at baseline and 9 months.

**Table 2:**
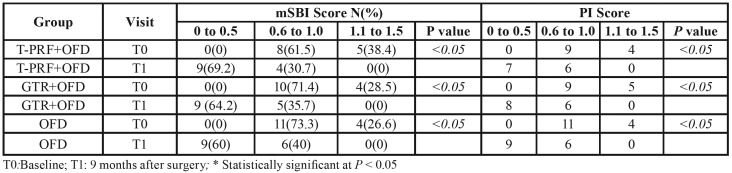
mSBI and PI in Groups at Baseline and 9 Months.

## Discussion

Endo-perio lesions are cases that require detailed diagnosis and planning in terms of both periodontal and endodontic lesions that are difficult for the clinician to overcome. The choice of the right regenerative periodontal treatment approach plays an essential role in the success of the treatment of these cases that require a multidisciplinary treatment approach (2,4). Therefore, in this study, the effect of different regenerative periodontal treatment approaches on healing was compared in IBDs with endo-perio lesion (primary perio-secondary endodontic lesion or true combined lesion). 

In the literature, many studies used bone graft materials and membranes together (18) and PRF alone or in combination with graft materials (19) in regenerative periodontal treatment of teeth with IBDs. Natural fibrin is formed in the first phase of flap healing process following OFD. It has been reported that T-PRF contributes to GTR by remaining in situ for one month without resorption of the tissue due to the formation of a denser and tighter fibrin scaffold (20). Besides, T-PRF is a preferable autogenous healing material that needs only the blood of the patient and has no costs at all (10). Therefore, in the present study, we aimed to use naturally biomimetic methods and observed the regenerative effect of T-PRF. Previous reports in the literature have been conflicting regarding the use of platelet concentrates alone or in combination with bone replacement grafts. While some researchers concluded superior clinical effectiveness in favor of the combined use (21), some others demonstrated that it did not confer any additional advantage (22). In the present study, we used T-PRF alone as a graft and membrane material in the experimental group and evaluated its effect on periodontal regeneration. 

In the success of regenerative treatment, tooth morphology (single or multiple roots), defect topography, and flap designs play an essential role (23). Molar teeth were not included in the study due to difficulties in surgical access, complicated root canal morphology and furcation defect. For this reason, single root and single canal teeth were included in the study with the intention of standardization. In the literature, it has been reported that there is a positive correlation between the success of regenerative procedures and the remaining number of bony walls (24). For this reason, we saw fit not to include one-wall defects in this study. Besides, some various flap designs such as conventional flap, papilla preservation flaps, and minimally invasive surgical approaches with or without papilla elevation have been observed to prevent exposure of regenerative biomaterials and reduce the risk of early wound healing failure in regenerative procedures (16,25). Schincaglia *et al*. (16) researched single versus double flap approach in periodontal regenerative treatment and concluded that any significant differences were not found between groups at 6-months, as for changes in PD and radiographic defect fill. In our study, we performed double flap approach to access both buccal and oral sides of tooth defects, and this approach did not cause any exposure of regenerative biomaterials. 

In most researches, periodontal clinical parameters and intraoral periapical (IOPA) radiographs are used for the assessment of healing and bone filling (15,26). In our study, we compared the effects of T-PRF and GTR on IBDs by clinical parameters, including PD, CAL, sulcus bleeding, and plaque index and periapical radiographs. According to our results, a decrease was observed in the 9th month in PD, CAL and IBD depth in all groups compared to the baseline parameters. Consistent with our results, Thorat *et al*. (27) and Pradeep *et al*. (19) found a decrease in PD, CAL values in all groups when compared with baseline values. That decrease may be explained by reduction of CAL which might have been the result of the formation of new attachment in case of PRF or T-PRF groups, and healing by repair, which indicates the presence of a long junctional epithelium between the newly regenerated tissues and the root surface in case of OFD.

In our study, the GTR and T-PRF groups showed a further decrease in PD and IBD depth compared to the OFD alone group after nine months of follow up. Similarly, Thorat *et al*. (27) investigated the clinical effects of PRF in the treatment of IBDs and demonstrated a greater decrease in PD and greater CAL gain and IBD fill in sites treated with PRF compared to their OFD group. Besides, Pradeep *et al*. (19) assessed platelet-rich plasma and PRF in the treatment of 3-wall IBDs and reported similarities in PD decrease, CAL gain, and bone fill in sites treated with PRF or PRP combined with OFD. They also reported that PRF was less time-consuming and less technique-sensitive, suggesting that it may be a better treatment option than PRP (28). In the preparation of T-PRF, blood is activated with titanium. Compared to L-PRF, PRF leads to a more mature and aggregated form, a firmer network structure, and may last a bit longer in the tissue (10,20). The fibrin releases various growth factors and stimulates the migration of tissue-forming cells, such as the fibroblasts and endothelial cells involved in angiogenesis and differentiation in the osteoblasts, therefore enhancing wound healing and periodontal regeneration (29). A further reduction in PD and IBD depth values ​​in T-PRF compared to OFD alone can be explained by these properties.

 Also, no difference was found between the T-PRF and GTR groups in terms of PD and CAL, while the GTR group was found to have a greater decrease in IBD depth. On the contrary, some studies compared the demineralized freeze-dried bone allograft (DFDBA) and PRF treatment options in IBD treatment and obtained no statistically significant difference between the groups in terms of PD, CAL and IBD filling at six months (7,26). Differences between study results may be due to methodological differences such as tooth selection, the morphology of the defect, regenerative treatment approach, follow-up time. Besides, further reduction in IBD depth in the GTR group compared to T-PRF can be explained by the fact that the graft materials resorb later and have greater radiopacity than T-PRF. On the other hand, no difference between the T-PRF and GTR groups in terms of PD and CAL may be explained by the mechanical adhesive properties of fibrin contributing to the stabilization of the flap and the proliferation of growth factors and neoangiogenesis (30).

Conclusion

According to our results, a treatment approach using T-PRF and GTR was more effective than one using OFD alone in the treatment of IBDs with endo-perio lesions. Also, as an easy-to-prepare biomaterial that is T-PRF was a successful alternative to graft materials in the treatment of IBDs. However, we believe that further studies with long-term follow-ups and histological and immune-histochemical data are still needed.
